# Multi-Site Spinal Cord Transcutaneous Stimulation Facilitates Upper Limb Sensory and Motor Recovery in Severe Cervical Spinal Cord Injury: A Case Study

**DOI:** 10.3390/jcm12134416

**Published:** 2023-06-30

**Authors:** Pawan Sharma, Tudor Panta, Beatrice Ugiliweneza, Robert J. Bert, Yury Gerasimenko, Gail Forrest, Susan Harkema

**Affiliations:** 1 Kentucky Spinal Cord Injury Research Center, University of Louisville, Louisville, KY 40202, USA; tudor.panta@uoflhealth.org (T.P.); beatrice.ugiliweneza@louisville.edu (B.U.); yury.gerasimenko@louisville.edu (Y.G.); susan.harkema@louisville.edu (S.H.); 2 Frazier Rehabilitation Institute, University of Louisville Health, Louisville, KY 40202, USA; 3 Department of Health Management and Systems Science, University of Louisville, Louisville, KY 40202, USA; 4 Department of Neurological Surgery, University of Louisville, Louisville, KY 40202, USA; 5 Department of Radiology, University of Louisville, Louisville, KY 40202, USA; robert.bert.1@louisville.edu; 6 Department of Physiology, University of Louisville, Louisville, KY 40292, USA; 7 Pavlov Institute of Physiology, Russian Academy of Sciences, 199034 Saint Petersburg, Russia; 8 Department of Physical Medicine & Rehabilitation, Rutgers New Jersey Medical School, Newark, NJ 07052, USA; gforrest@kesslerfoundation.org; 9 Kessler Foundation, Newark, NJ 07052, USA; 10 Department of Bioengineering, University of Louisville, Louisville, KY 40202, USA

**Keywords:** cervical spinal cord injury, upper limb, spinal cord transcutaneous stimulation, neuromodulation, spinal cord excitability, motor rehabilitation

## Abstract

Individuals with cervical spinal cord injury (SCI) rank regaining arm and hand function as their top rehabilitation priority post-injury. Cervical spinal cord transcutaneous stimulation (scTS) combined with activity-based recovery training (ABRT) is known to effectively facilitate upper extremity sensorimotor recovery in individuals with residual arm and hand function post SCI. However, scTS effectiveness in facilitating upper extremity recovery in individuals with severe SCI with minimal to no sensory and motor preservation below injury level remains largely unknown. We herein introduced a multimodal neuro-rehabilitative approach involving scTS targeting systematically identified various spinal segments combined with ABRT. We hypothesized that multi-site scTS combined with ABRT will effectively neuromodulate the spinal networks, resulting in improved integration of ascending and descending neural information required for sensory and motor recovery in individuals with severe cervical SCI. To test the hypothesis, a 53-year-old male (C2, AIS A, 8 years post-injury) received 60 ABRT sessions combined with continuous multi-site scTS. Post-training assessments revealed improved activation of previously paralyzed upper extremity muscles and sensory improvements over the dorsal and volar aspects of the hand. Most likely, altered spinal cord excitability and improved muscle activation and sensations resulted in observed sensorimotor recovery. However, despite promising neurophysiological evidence pertaining to motor re-activation, we did not observe visually appreciable functional recovery on obtained upper extremity motor assessments.

## 1. Introduction

Approximately sixty percent of spinal cord injuries (SCI) occur at the cervical cord level, resulting in partial or complete loss of sensory and motor function in the upper extremity, trunk, and legs, severely affecting the independence and quality of life [1]. Individuals with cervical SCI rank regaining arm and hand function as their top rehabilitation priority, often even more desired than the other functions, such as walking, bladder and bowel control, and sexual function [2,3]. To perform essential activities of daily living including personal hygiene, eating, and grooming, optimal functioning of the upper extremity is required. Therefore, even a small functional gain in the upper extremity function can greatly enhance their quality of life and independence [4].

Studies on the use of epidural spinal cord stimulation have shown to be effective in recovering various physiological functions, such as voluntary activation of upper [5] and lower limb muscles [6], independent stepping [7,8], postural stability [9,10], and assisted ambulation in humans post SCI [11,12]. Recent findings suggests that similar to epidural stimulation, non-invasive spinal cord transcutaneous stimulation (scTS) can target specific motor neuronal pools in the spinal cord [13,14] and primarily exerts its physiological effects via activation of medium-to-large size sensory afferents [13,15]. Drawing inspirations from the therapeutic effectiveness of lumbosacral scTS in facilitating neuromotor functions [16,17,18,19], scTS effectiveness in restoring lost upper extremity function post cervical SCI has received considerable scientific and clinical attention in the recent years. More specifically, chronic application of cervical scTS combined with motor rehabilitation training is shown to be effective in recovering sensory and motor function post-cervical SCI [20,21,22,23,24]. 

Although promising, the majority of the work on scTS has included participants with incomplete SCI or participants with residual arm and hand function measured using various motor tasks and neurophysiological assessment [20,21,22,23,24]. Moreover, recent findings suggest that scTS augmented motor recovery is prominently seen in individuals with residual motor functions post cervical SCI [14,25], raising the following important and clinically relevant question: does the chronic application of cervical scTS combined with activity-based recovery training (ABRT) facilitate recovery of sensory and motor function in individuals with severe SCI who have minimal to no preservation of upper extremity sensory and motor function? 

To answer this question and obtain proof-of-principle data, we conducted a study involving a 53-year-old male with C2 level (American Spinal Injury Association Impairment Scale (AIS)-A, 8 years post-injury), who underwent 60 sessions of scTS targeting various spinal segments combined with ABRT. Each session lasted for approximately 60 min. The spinal segments and stimulation parameters were identified using comprehensive spinal cord excitability and motor task specific mapping. We hypothesized that the employed multimodal neuro-rehabilitative approach involving multi-site scTS will neuromodulate the spinal networks and enhance the activity-dependent neuroplastic effects of ABRT, resulting in sensory and motor recovery in participants with severe cervical SCI. Neurophysiological and functional outcomes were obtained pre, mid and post 60 ABRT sessions. We herein demonstrate that ABRT combined with targeted multi-site scTS, is effective in enabling sensory and motor recovery in a participant with severe cervical SCI. Interestingly, greater improvements in sensory and motor function were observed in the presence of scTS. Most likely, altered spinal cord excitability and improved muscle activation and sensations can potentially explain the neurophysiological basis for the observed recovery. However, despite promising neurophysiological evidence pertaining to motor re-activation, we did not observe visually appreciable functional recovery on obtained upper extremity motor assessments. The obtained findings of the present study contribute to extending the therapeutic effectiveness of multi-site scTS combined with ABRT to individuals with severe cervical SCI, and will aid in the development of appropriate neuromodulation and rehabilitation programs for those with debilitating motor and sensory dysfunctions post cervical SCI.

## 2. Materials and Methods

### 2.1. Clinical Characteristics of the Participant

**Figure 1:**
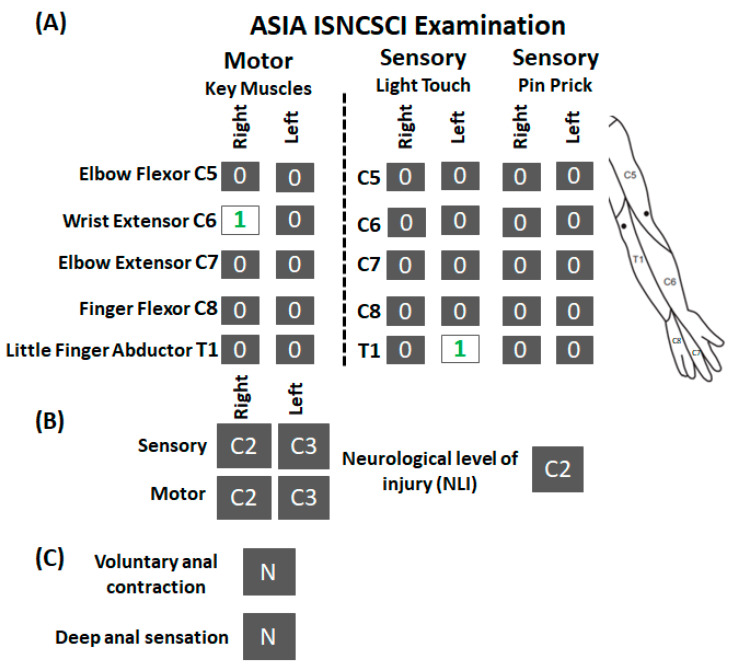
ISNCSCI examination for research participant A134. (**A**) Shown are the scorings of ISNCSCI assessment for motor and sensory (light touch and pin prick) components along with the schematic of dermatomes location tested during the assessment. (**B**) Neurological level of injury for motor and sensory component on the right, left side, and overall. (**C**) Status of anal contraction and sensations. As seen, except right wrist extensor muscle and left T1 light touch (numericals in green), the participant scored zero on all motor and sensory subcomponents. ISNCSCI: International Standards for Neurological Classification of Spinal Cord Injury; N: No.

The participant (A134) was a 53-year-old male who experienced a cervical SCI secondary to fall during skiing eight years ago. He underwent spinal fusion surgeries for C4–C6 vertebral stabilization. The American Spinal Cord Injury Association (ASIA) International Standards for Neurological Classification of SCI (ISNCSCI) [26] examination revealed that he suffered a clinically complete SCI (AIS A) and his overall neurological level of injury was C2 (Figure 1). More specifically, all key upper extremity muscles, except the wrist extensor on the right side, scored zero for the right and left sides. Similarly, all key upper extremity dermatomes (C5–T1), scored zero for light touch and pinprick for both sides, except T1 light touch on the left, which scored 1 (impaired sensations). Before enrolling in the study, he underwent physical rehabilitation programs mainly focusing on lower limb function, such as step training and functional electrical stimulation during biking. He never underwent a dedicated rehabilitation training program targeting upper extremity functions. At the time of enrollment, he was operating a sip and puff based powered-wheelchair and required 100% assistance during activities of daily living. The experimental protocol was approved by the Institutional Review Board (IRB# 21.0588) at the University of Louisville and was in accordance with the Declaration of Helsinki. The participant provided written informed consent before participating in the study.

### 2.2. Magnetic Resonance Imaging (MRI) Data Acquisition

The sagittal and axial view of the cervical and thoracic spinal cord was collected using 2-D described elsewhere [27,28]. Briefly, images were obtained using a 3 Tesla system (Siemens Magnetom Skyra, Siemens Medical Solutions, Malvern, PA, USA) with Turbo Spin Echo T2-weighted pulse sequences from the foramen magnum to the thoracic regions. Sagittal images were obtained in two or three sequences with large field of view (FOV) images to screen patients for syringes, significant stenoses, scoliosis, levels of injury and stabilizing treatment-related surgical changes. Typical parameters were:

TR/TE/FA/Thick/ETL/Re-Matrix/PFOV/NSA/BW/Pixal/AQ-matrix/%samp/PE=

Upper sagittal:

3000/74/160/3 × 3.45/17/320 × 320/100%/2/600/1.125 × 1.125/320 × 240/75/442

Lower Sagittal:

3000/74/~130/3 × 3.45/17/320 × 320/100/2/600/1.25 × 1.25/324 × 240/75/442

where TR = repetition time; TE = echo time; ETL = echo train length; Re-Matrix = reconstruction matrix; PFOV = %phase field of view; NSA = number of signal averages; BW = bandwidth; Pixal = pixel dimensions; AQ-matrix = acquisition matrix; % samp = % sampling (or partial fourier) and PE = number of phases 3 encodes.

T2 weighted axial images were obtained using 3 mm thickness slices with 3 mm gap between consecutive slices. The example parameters used were as follows:

Cervical spine:

5190/74/160/3 × 3/26/512 × 512/100/2/610/0.35 × 0.35/256 × 179/70/260

Thoracic spine:

3690/82/121/5 × 10/28/512 × 512/100/2/610/0.35 × 0.35/256 × 179/70/252

### 2.3. Functional Neurophysiological Assessment (FNPA)

The participant underwent an FNPA to investigate the neurophysiological evidence of motor activity in selective upper extremity muscles [29,30]. Briefly, in supine position, the skin was prepared for electrode placement by shaving and cleaning with an alcohol pad. Pairs of bipolar surface EMG electrodes were placed mid-belly on the following muscles bilaterally: biceps brachii (BB), triceps brachii (TB), extensor carpi radialis (ECR), flexor capri radialis (FCR), extensor digitorum profundus (EDP), flexor digitorum profundus (FDP), abductor digiti minimi (ADM), first dorsal interossei (FDI), lateral trapezius (Lat. Trapz.), medial trapezius (Med. Trapz.), anterior deltoid (Ant. Del.), and lateral deltoid (Lat. Del.) [29]. The location of electrode placement for each muscle was selected based on the standard SENIAM and other guidelines [31,32]. All research staff received significant training to reliably place electrodes over specific muscles location and avoiding differences in the electrode placement between assessment sessions [29]. The participant was instructed to perform controlled and isolated three 3-s movement trials with full force, termed “events”, for each muscle against manual resistance resulting in no visible movement of the target upper extremity segments (BB: elbow flexion; TB: elbow extension; ECR: wrist extension; FCR: wrist flexion; EDP: finger extension; FDP: finger flexion; ADM: little finger abduction; FDI: index finger adduction; Lat. Trapz: shoulder-shrugging; Med. Trapz: shoulder-shrugging; Ant. Del.: shoulder flexion; Lat. Del.: shoulder abduction). A minimum of 3–10 s rest period was provided between two successive events. An audible tone of three seconds was used to cue the participant: the start and end of the tone signaled the participant to begin and cease the event, respectively. The time points for the start and end of the tone were automatically registered by the data collection setup, and an experienced examiner instructed the participant to perform the selected upper extremity movements. The EMG signals were recorded using a custom-written acquisition software (National Instruments, Austin, TX, USA) with a sampling rate of 2 kHz per channel and a bandpass of 30 Hz to 2 kHz (MA400, Motion Labs Systems, Baton Rouge, LA, USA).

### 2.4. Multisegmental Motor Response (MMR) Mapping

The participant underwent a comprehensive MMR mapping to investigate the intrinsic excitability of the spinal cord in recruiting various upper extremity muscles in response to scTS targeting various spinal levels. Briefly, in the supine position, the skin preparation and EMG electrode placement was performed as described for the FNPA [29]. To stimulate cervical and thoracic spinal levels, seven 2.5 cm diameter hydrogel adhesive electrodes (3.175 cm diameter, PALS^®^, Axelgaard, Fallbrook, CA, USA) were applied to vertebral levels C3–C4, C4–C5, C5–C6, C6–C7, C7–T1, T1–T2 and T11–T12 midline as cathodes in consecutive order to avoid overlapping between the stimulating electrodes. The anodes or returning electrodes (5.08 cm × 8.89 cm diameter, PALS^®^, Axelgaard, CA, USA) were applied to the bilateral clavicle. Stimulation was delivered using a constant current stimulator (DS8, Digitimer, Welwyn Garden City, UK) with an unmodulated 1 ms duration monophasic rectangular pulse at 0.2 Hz. The stimulation intensity was increased in an interval of 5 mA, starting from 5 mA to 150 mA. The resulting MMRs were recorded using Spike2 software (CED Ltd., Cambridge, UK) at 2 kHz sampling rate (MA400, Motion Labs Systems, Baton Rouge, LA, USA) and saved for the offline analysis. Additional care was taken to ensure that the participant was comfortable throughout the session and stimulation did not induce any adverse effects, such as autonomic dysfunction and skin irritation/burns. 

### 2.5. Upper Extremity Volitional Movement Ability Mapping

The participant underwent a comprehensive voluntary movement mapping to identify scTS effects on the upper extremity movements. In addition to the spinal level identified based on the MMR mapping, we also investigated the effect of T11–T12 spinal level stimulation on the upper extremity motor performance as stimulating this site is known to modulate the excitability of the cervical spinal cord [33,34] and is also effective in improving trunk control [35,36,37] which is required for the successful execution of upper extremity tasks.

**Figure 2:**
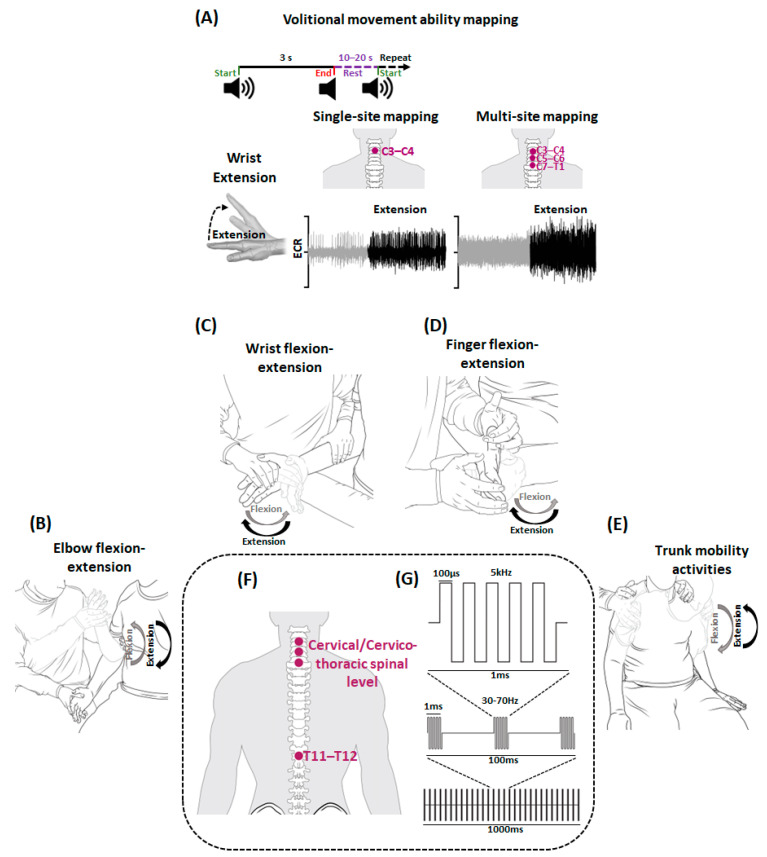
Schematics for Volitional movement ability mapping and activity-based recovery training (ABRT) combined with spinal cord transcutaneous stimulation (scTS). Shown is the schematic for (**A**) Volitional movement ability mapping. In sitting position, participant was instructed to perform various upper extremity movements during a three-second audio cue (wrist extension is shown in the illustration) with scTS during single-site and multi-site mapping. The obtained EMG during rest (gray) and activity (black) from prime mover (ECR) is stored for offline analysis. A rest period of 10–20 s is provided between consecutive events. Shown are the schematics representing (**B**) elbow flexion-extension (**C**) wrist flexion-extension (**D**) finger flexion-extension (**E**) trunk activities practiced during a typical ABRT session. In sitting position (**F**) stimulating electrodes targeting cervical and thoracic spinal segments were used to deliver (**G**) rectangular biphasic, 1 ms pulse duration with 5 kHz modulation pulses (adapted from [38]). Note that the schematics are for illustration purposes and not drawn to the scale. ECR: Extensor Carpi Radialis.

During a typical mapping session, after placing EMG electrodes to the selected upper extremity muscles, the participant was transferred to a mat and maintained an upright sitting posture with adequate support from the research technician. To investigate elbow flexion control, the participant’s forearms were fully supported on an adjustable table in pronated position. In contrast, for wrist extension, a roll of sheet was placed beneath the wrist to allow the wrist to be in slight flexion so that any appreciable wrist extension can be easily identified by the examiner. The participant was instructed to perform a controlled and isolated three 3-s movement trials involving bilateral elbow flexion or wrist extension with full force along with the audio tone. At the end of three movement trials, the participant was asked to grade the movement performance as no-change, positive, or negative compared to the previously performed movement trials. Visually appreciable features, such as muscle twitching, visible movement, and subjective reporting, such as tingling sensations in the targeted upper extremity, feeling of more connected or ease while attempting to move the body segment, were considered as positive effects of the stimulation. In contrast, feelings, such as difficulty in attempting the movement trial, fatigue or decrease in movement performance, were reported as negative effects. No difference compared to the previously performed movement trials was reported as no change. A minimum of 10–20 s rest period was provided between 3 successive movement trials (Figure 2A).

The mapping assessment was performed in two phases: Single-site and Multi-site mapping. During single-site mapping, for each stimulation site, amplitude and frequency mapping was performed. During amplitude mapping, stimulation intensity was gradually increased in a step of 10 mA from 20 mA to 90 mA or maximum tolerable intensity for all sites except T11–T12 where maximum stimulation intensity was set to 120 mA. Throughout, stimulation frequency was maintained at 30 Hz. Similar stimulation intensities and frequencies have been used previously [20,22]. The stimulation intensities at which the participant or examiner reported positive effects were compared to intensities at which the participant reported negative effects or no change to further ascertain the accuracy of participant’s/examiner’s reporting. The stimulation intensity at which the participant reported consistently positive motor performance was noted to be used later during the frequency mapping. During frequency mapping, for each stimulation site, the stimulation intensity identified during amplitude mapping was used at varying frequencies from 30 to 70 Hz in an interval of 5 Hz. Frequencies at which the participant or examiner reported consistent positive effects were ascertained using methodology opted for amplitude mapping.

During multi-site mapping, the stimulation intensities and frequencies identified for each stimulation site during single-site mapping were simultaneously used in different combinations. For each combination, the stimulation frequency was set at the pre-determined levels and amplitude was ramped up for the distal followed by proximal stimulation sites. For example, in C3–C4 + T11–T12 combination, the T11–T12 stimulation site was ramped up first, followed by the C3–C4 site. The stimulation intensity was increased until the pre-determined or maximum tolerable stimulation levels were reached. The participant was then instructed to perform movement trials and movements were categorized as positive, negative or no change based on the participant’s or examiner’s feedback. As the participant experienced severe SCI with no appreciable motor activity on the visual inspection, subjective reporting during the mapping sessions was primarily considered while making decisions about the optimal stimulation intensity and frequency. 

### 2.6. Activity-Based Recovery Training (ABRT)

All ABRT sessions were performed with the participant seated on the mat with adequate trunk support as needed and hips and knees at 90°. The goal of each training session was to improve volitional contraction capabilities by engaging various upper extremity segments in active assisted range of motion (Figure 2B–E). During training, the participant was encouraged to visualize and volitionally engage in the entrusted upper extremity movement (described later) while being assisted by the therapist for entire range of motion. Verbal and tactile cueing was provided as needed and care was taken to avoid compensatory behavior when attempting upper extremity movements, such as mitigating ipsilateral scapular elevation when attempting finger flexion. Both upper extremities were engaged in below described tasks alternately to avoid fatigue for 60 min with scTS. 

The participant performed 7.5 min of elbow flexion followed by 7.5 min of elbow extension with 8–10 min of rest with no scTS between them. This was followed by 5 min of wrist and fingers flexion or extension. To allow adequate number of repetitions for wrist and finger activities, flexion and extension activities were performed on the alternate days. The session ended with 10 min of trunk mobility activities. For elbow flexion and extension, stabilization was provided at the distal end of the humerus (Figure 2B). To allow maximum activation of the biceps brachii, elbow flexion was performed against gravity with the forearm in a supinated position. Additionally, from 30–60 ABRT sessions, elbow extension movements were performed in the gravity-eliminated plane with the forearm and wrist pronated and positioned over a tabletop below chest level in the frontal plane. A pillow sheet was placed under the participant’s forearm and wrist to minimize friction. Due to participant presenting with bilateral glenohumeral subluxation, the trainer ensured manual glenohumeral stabilization for appropriate centration during any humeral elevation. For wrist and finger movements, the participant was positioned with the shoulder in neutral, elbow flexed to 90° and forearm in neutral. During wrist movements, stabilization was provided proximal to the wrist joint. In contrast, stabilization was provided over the carpal bones during finger movements (Figure 2C,D). During trunk movements, the participant performed two sets of 10 repetitions of trunk flexion, extension, and right and left lateral trunk flexion (Figure 2E).

### 2.7. Spinal Cord Transcutaneous Stimulation (scTS)

To effectively modulate the spinal circuitry pertaining to upper extremity function, scTS was delivered during ABRT sessions targeting different spinal levels using stimulation parameters identified during the volitional movement ability mapping (Figure 2F). scTS was delivered using a BioStim-5 stimulator (Cosyma Inc., Denver, CO, USA) using rectangular, biphasic, 1 ms pulse duration with 5 kHz modulation (Figure 2G). Active electrodes (cathode, 3.175 cm diameter, PALS^®^, Axelgaard, CA, USA) were placed at specific spinal segment levels and the common returning electrodes or anodes (5.08 cm × 8.89 cm diameter, PALS^®^, Axelgaard, CA, USA) were placed on the clavicles or iliac crest depending on the stimulation site. For each stimulation site, at pre-determined frequency, the stimulation intensity was gradually increased to the identified levels or the maximum tolerable intensity without discomfort. A rest of period 8–10 min with no stimulation was provided every 20 min to avoid muscle fatigue and potential redness of the skin and was not included in the training duration. Unlike other studies that positioned anodes over the iliac crest for cervical spinal cord stimulation [22,23], anodes were placed on the clavicle as we observed greater MMR amplitudes during scTS targeting different spinal levels when anodes were placed at clavicle compared to the iliac crest (Supplementary Figure S1A). As we intended to modulate the spinal cord excitability using multi-site scTS without causing direct activation of the target upper extremity muscles, the selected stimulation intensity was subthreshold in nature [22]. Indeed, single-site stimulation using parameters identified during voluntary control mapping did not evoke MMRs indicating that the stimulation was subthreshold in nature (Supplementary Figure S1B,C).

### 2.8. Upper Extremity Motor Assessment

Upper extremity gross and fine motor performance was assessed using standardized Graded Redefined Assessment of Strength, Sensibility, and Prehension (GRASSP) assessment. The details related to the assessment procedure and its anthropometric properties can be found here [39]. Briefly, for each upper extremity, the participant was assessed on different aspects including muscle power, sensation, quality of grasping patterns, and time required to perform various fine and gross upper extremity tasks. The assessment was performed during pre-training, post 20, 40, and 60 training sessions with and without scTS using stimulation sites and parameters identified during the volitional movement ability mapping. 

Additionally, the participant’s ability to perform various tasks involving the upper extremity and trunk was assessed using neuromuscular recovery scale (NRS) [40,41]. NRS evaluates an individual’s ability to perform motor tasks post SCI in relation to pre-injury functional capabilities. Nine sub-items of NRS focusing on upper extremity and trunk function, including overhead press, shoulder flexion, grasp, door pull, open with key, can open and manipulation, sit up, trunk extension in sitting, and door pull and open, were collected. Items were scored from phase 1 to phase 4. The higher the phase, the better the functional performance. Incremental performance within each phase was denoted using the alphabet A-C. All functional assessments were performed by a registered occupational/physical therapist trained to acquire the described assessments.

### 2.9. Data Analysis

All EMG data were analyzed using custom-written MATLAB programs (Version 2021a, MathWorks Inc., Natick, MA, USA). The EMG data during each event during FNPA or movement trial during volitional movement ability mapping were extracted using the cue markers. The data was then demeaned, band-pass filtered (25–900 Hz, Butterworth filter, second order) and notch filtered (60 Hz). Spectral analysis of EMG data obtained during volitional movement ability mapping was performed using conventional or short FFT with hamming window equal to the length of the movement trial (3 s). To obtain Δ change, EMG activity during the event (3 s) was normalized using the EMG activity during the rest period (3 s) prior to each attempt. No such digital signal processing was performed for MMR data. 

MRI analysis details have been previously defined [27,42,43]. Briefly, all T2-weighted images were analyzed using the open-source Spinal Cord Toolbox (Version 5.1). For intact spinal cord, the toolbox automatically segmented the region of interest (ROI) of the spinal cord using in-built machine learning tools for the axial slices. The ROIs per slice were visually inspected for the quality check. In case the segmented ROIs did not match with the actual spinal cord contour, appropriate changes were made using open-source FSLeyes software (FSL 5.0.10). In contrast, for lesioned spinal cord, lesion boundaries were created using a manually drawn mask based on the lesion hyperintensity and its contrast from the surrounding intact spinal cord using FSLeyes software. As vertebral level identification was easier on the sagittal view than the axial view, the sagittal images were co-registered with axial images to obtain level-specific outcomes, such as injured and non-injured spinal cord cross-sectional area, diameter, and solidity [42]. All ROIs were checked by a board-certified radiologist (American Board of Radiology, USA) with a subspecialty Certificate of Added Qualification in Neuroradiology (American Board of Radiology) and 20 years of experience in neuroradiology in both research and clinical practice (R.J.B.).

To obtain spinal level excitability ranking, the obtained MMRs were first extracted and assessed for signal quality. Responses that appeared noisy or aberrant were rejected and excluded from the final analysis. For analysis, a 100 ms window after the stimulation was assessed to identify the actual evoked response to the stimulus. Generally, MMRs were seen between 10–35 ms time window. Mean peak-peak (P2P) amplitude was obtained for minimum three individual responses at each stimulation intensity. For each muscle, the obtained mean P2P amplitude at different stimulation intensities and across stimulation sites was tabulated to find the maximum P2P amplitude. The obtained maximum P2P amplitude was later used to normalize the tabulated mean P2P amplitude to obtain normalized amplitudes ranging from 0–1 across all stimulation sites. For each muscle, the sum of normalized scores from all stimulation intensities was compared across stimulation sites, and the spinal level excitability ranking was determined. 

Normalized recruitment curves and selectivity index of MMRs were obtained for the selected upper extremity muscles in responses to scTS targeting different spinal levels using the method previously defined [44]. Briefly, for each stimulation site (*s*tim), muscle (mus) and stimulation intensity (int), normalized mean and standard deviation of *P*2*P* amplitude of MMRs were denoted as $RC_{stim,mus}$ and $stdRC_{stim,mus}$, respectively, and obtained using the following formulae:

$$RC_{stim,mus}=\frac{P2P_{stim,mus}}{max_{stim^{\prime },int’}\left\{P2P_{stim^{\prime },mus}\left(int’\right)\right\}}\;stdRC_{stim,mus}=\frac{sP2P_{stim,mus}}{max_{stim^{\prime },int’}\left\{P2P_{stim^{\prime },mus}\left(int’\right)\right\}}$$

The selectivity index (SI) for muscle (mus) was computed as:

$$SI_{stim,mus}=P2P_{amp\epsilon DR}\{\begin{array}{c} RC_{stim,mus} \end{array}\left(amp\right)-\frac{1}{Namps-1}\underset{M’\neq M}{\sum }RC_{stim,mus^{\prime }}\left(amp)\right\}$$

where *DR* denotes the dynamic range of stimulation intensities and includes the mean of *P*2*P* from threshold amplitude (muscular recruitment > 10% in at least one muscle) to saturation amplitude (muscular recruitment > 90% in every muscle or at the highest stimulation intensity). 

### 2.10. Statistical Analysis

As the presented work is of a case study nature, statistical analysis is limited. However, the statistical significance of differences in EMG change during FNPA and MMR P2P amplitude pre and post-sixty ABRT sessions was calculated using 2-sided Paired *t*-test following a non-violation of normality assumption per the Kolmogorov-Smirnov test. All statistical analyses were performed using Microsoft Excel (IBM Corp., Armonk, NY, USA), MATLAB (Version 2021a, MathWorks Inc., Natick, MA, USA) and SAS 9.4 (SAS Inc., Cary, NC, USA). All data is presented as mean ± SD, else otherwise noted. The statistical significance threshold was set at 0.05. 

## 3. Results

### 3.1. Severity of Injury

**Figure 3:**
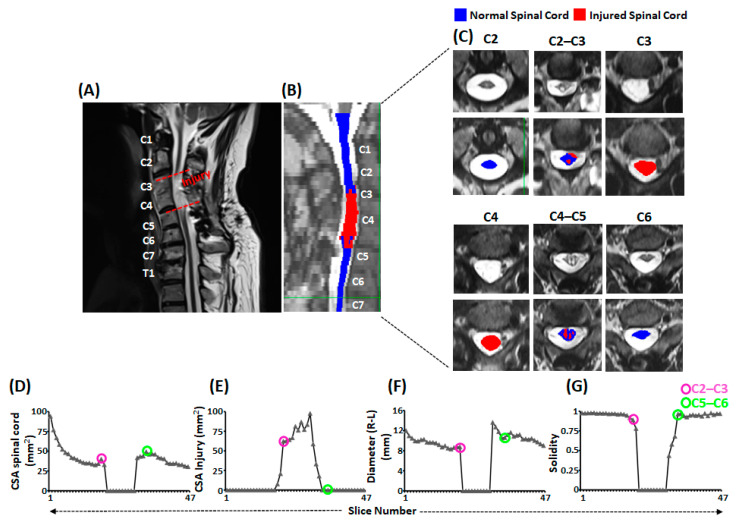
Morphometric analysis of the injured spinal cord. Shown is (**A**) the sagittal view of T2-weighted MRI of cervical and thoracic spinal cord with injury confined between dashed red lines (**B**) the sagittal view of T2-weighted image of cervical and thoracic spinal cord with overlaid masks representing normal (blue) and injured (red) spinal cord segments. The vertebral body levels are indicated with texts in white. (**C**) Shown are the axial slices of the T2-weighted image of cervical spinal cord from C2 to C6 vertebral levels without (top panel) and with (lower panel) overlaid masks representing normal (blue) and injured (red) spinal cord. (**D**–**G**) line plots representing various morphometric aspects of the spinal cord to indicate the severity of injury. The pink and green circled points on the line plots correspond to the C2–C3 and C5–C6 intervertebral discs level, respectively. MRI: Magnetic Resonance Imaging; CSA: Cross-sectional Area.

Spinal cord MRI images and quantification of the cross-sectional area of the injured and spared spinal cord revealed the severity of the injury (Figure 3). On visual inspection of the injured spinal cord (Figure 3A) and associated masks on the sagittal view (Figure 3B), SCI spanned from C3 vertebral upper border to C5 vertebral upper border. The axial images of the spinal cord revealed that the cord was injured as high as C2–C3 intervertebral level and extended as low as C5 vertebral level (Figure 3C). Moreover, quantification of the axial images revealed that compared to the cross-sectional area of the intact spinal cord, there was a drastic reduction in the spinal cord area at C3 and C4 vertebral levels (Figure 3D). In contrast, cross-sectional area of the injured spinal cord demonstrated a drastic increase between C3 to C5 vertebral levels, with a maximum increase around the C4 vertebral level and no viable spinal cord tissue at C3 and C4 vertebral levels (Figure 3E). In addition to the cross-sectional area loss, the cord also suffered compression in the mediolateral directions (Figure 3F). The combination of CSA loss and mediolateral compression disturbed the overall architecture of the spinal cord, as evidenced by the solidity score of less than 1 (1 = elliptical shape, <1 = disturbed elliptical shape) (Figure 3G).

### 3.2. Activation of Upper Extremity Muscles via scTS

**Figure 4:**
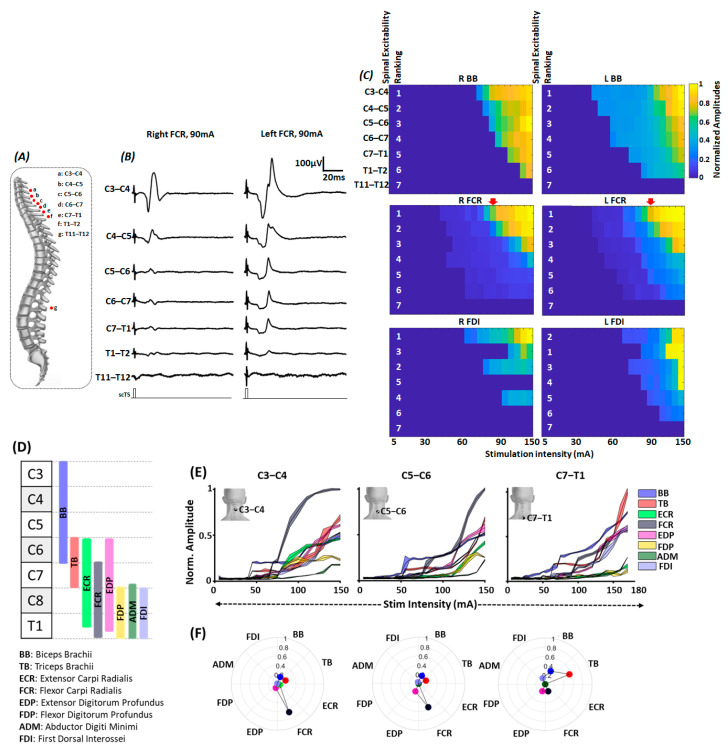
Multisegmental motor response (MMR) mapping. (**A**) Schematic to represent the approximate position of stimulating electrodes in relation to the spinous levels during MMR acquisition. (**B**) Shown are the representative MMR evoked in FCR muscle secondary to the stimulation at different spinal levels with 90 mA intensity. (**C**) Shown are the heatmaps representing the normalized peak-peak amplitude for bilateral BB, FCR and FDI muscles secondary to the stimulation of different spinal levels at various stimulation intensities. The white numbers in heatmaps represent spinal level excitability ranking. Inverted red arrows represent the column of peak-peak amplitude for bilateral FCR shown in Figure 3B. As seen, stimulation targeting C3–C4 spinal level resulted in highest MMR amplitudes. (**D**) Schematic representing the approximate distribution of motoneuronal pool supplying various upper extremity muscles along the spinal cord [45]. (**E**) Normalized peak-peak amplitude (**F**) selectivity index of various upper extremity muscles during C3–C4, C5–C6 and C7–T1 spinal stimulation. For normalized peak-peak amplitude, the shaded region denotes the standard deviation with mean value represented by the line in between. scTS: Spinal Cord Transcutaneous Stimulation; BB: Biceps Brachii; TB: Triceps Brachii; ECR: Extensor Carpi Radialis; FCR: Flexor Carpi Radialis; EDP: Extensor Digitorum Profundus; FDP: Flexor Digitorum Profundus; ADM: Abductor Digiti Minimi; FDI: First Dorsal Interossei.

Figure 4A shows the schematic of the relative position of scTS electrodes targeting different spinal levels in relation to the spinous processes and the resulting MMRs from right and left flexor carpi radialis (FCR) muscle secondary to scTS delivered to different spinal levels at 90 mA (Figure 4B). As seen, among all stimulation sites, C3–C4 stimulation resulted in maximum P2P amplitude of the obtained responses on both sides. Figure 4C shows the heatmap for normalized P2P amplitude of representative muscles from the arm (biceps brachii, BB), forearm (Flexor Carpi Radialis, FCR), and hand (First Dorsal Interossei, FDI) region for the right and left side in response to scTS targeting various spinal levels at varied stimulation intensities along with their excitability rankings (numericals in white). Spinal excitability ranking for the majority of the arm, forearm and muscles (only BB, FCR and FDI are shown here) revealed that the stimulation targeting the upper four stimulation sites (C3–C4, C4–C5, C5–C6 and C6–C7) resulted in the maximum activation of these muscles, with greatest activation during C3–C4 stimulation. 

As the activation of different upper extremity muscles was observed secondary to the stimulation targeting C3–C4 to T1–T2 spinal segments, we decided to utilize three site stimulation that will allow the spanning of electrodes from C3–C4 to T1–T2 spinal levels and effectively recruit majority of upper extremity muscles. Given that the size of the stimulating electrode (3.175 cm diameter) slightly spanned over the spinal segments above and below the stimulation site and as we did not observe drastic differences in the excitability ranking between the neighboring stimulation sites (C3–C4 vs. C4–C5 ranking), every alternate stimulation site was selected for effective recruitment of various upper extremity muscles, i.e., C3–C4, C5–C6, C7–T1. Indeed, closer inspection of the recruitment properties of different upper extremity muscles revealed differential activation of these muscles during scTS different spinal levels (Figure 4D–F). 

Schematic shown in Figure 4D represents the approximate rostro-caudal distribution of the motoneuronal pool innervating different upper extremity muscles in relation to the cervical spinal segments [45]. We expected to achieve greater activation of proximally innervated musing during C3–C4 and C5–C6 stimulation (BB, ECR, FCR, EDP, FDP) and distally innervated muscles during C7–T1 (TB, ADM and FDI) stimulation. As seen in the normalized recruitment curves for different upper extremity muscles secondary to C3–C4 stimulation, BB, TB were the first muscles to get activated around 40–60 mA. At higher stimulation intensities, FCR muscle demonstrated the greatest activation, and other muscles, such as BB, TB, ECR, EDP, FDP, and FDI, demonstrated similar activation profiles. Similarly, during C5–C6 stimulation, earlier activation of BB, TB, followed by FCR was observed. However, in contrast to C3–C4 stimulation, we observed reduced activation of ECR and FDP. Finally, during C7–T1, we observed least activation of ECR and FDP, and the greatest activation of TB and BB at higher stimulation intensities (Figure 4E).

Differential activation of various upper extremity muscles in response to scTS targeting different spinal levels was further corroborated by the findings of selectivity index (Figure 4F). For both C3–C4 and C5–C6 stimulation, the greatest activation was observed for the FCR muscle. Other muscles, such as BB, TB, demonstrated similar selective activation. Moreover, compared to C3–C4 stimulation, greater selective activation of EDP was observed during C5–C6 stimulation, with trends of decreased activation of FCR and ECR. In contrast, during C7–T1 stimulation, compared to proximal sites stimulation, as expected, we observed greater selective activation of TB and FDI muscles. Overall findings of the MMR mapping supported the notion that scTS targeting different spinal sites will allow differential activation of the various upper extremity muscles.

### 3.3. Greater Muscle Activation during Multi-Site Compared to Single-Site scTS

During volitional movement ability mapping, in addition to the stimulation sites targeting spinal levels to effectively recruit various upper extremity muscles (C3–C4, C5–C6, C7–T1), we also investigated the effects of individual and combinatorial T11–T12 stimulation as it is known to be effective in modulating the cervical spinal cord excitability [33,34] and enabling trunk stability needed to perform upper extremity functions [18,35,36].

**Figure 5:**
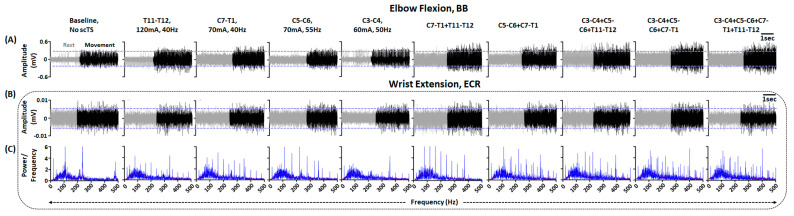
Upper extremity volitional movement ability mapping. Shown are the EMG activity in (**A**) BB and (**B**) ECR during elbow flexion and wrist extension, respectively, without scTs and with single or multi-site scTS at specific stimulation intensity and frequency. Upper and lower blue guidelines represent the EMG activity without scTS. (**C**) Shown are the FFT plots associated with ECR activity during wrist extension without scTs and with single or multi-site scTS. Red lines within FFT plots represent the moving mean of the obtained power at various frequencies. Note that during multi-site stimulation, similar parameters as single site stimulation were used. EMG: Electromyography; BB: Biceps Brachii; ECR: Extensor Carpi Radialis; FFT: Fast Fourier Transform; scTS: Spinal Cord Transcutaneous Stimulation.

As seen in Figure 5A, when instructed to perform elbow flexion without scTS, we observed a distinct muscle burst in the BB muscle. Compared to no scTS, stimulation targeting T11–T12 spinal segment resulted in a visually appreciable increase in the activation of the BB muscle. Similar facilitatory effects of single-site stimulation compared to the baseline muscle activity were observed for the remaining stimulation sites. Additionally, during T11–T12 stimulation, we observed greater upright sitting with the participant reporting improved trunk control. Compared to single-site stimulation, multi-site stimulation resulted in greater activation of BB muscle, with the greatest activation during stimulation targeting three (C3–C4 + C5–C6 + C7–T1) or four (C3–C4 + C5–C6 + C7–T1 + T11–T12) sites. In contrast, when instructed to perform wrist extension, we did not observe distinct activation of the ECR muscle with or without stimulation. However, spectral plot analysis demonstrated that scTS enhanced the firing frequency of the muscles to a degree comparable to that of a neurologically intact individual (smooth bell-shaped curve between 50–300 Hz and peak at approximately 100 Hz) (Figure 5B, Supplementary Figure S1D). Interestingly, greater activation with multi-site scTS was observed for the muscles with preserved functional activation, such as BB. However, in both conditions, we did not observe visually appreciable volitional upper extremity movements. Overall, the selected stimulation sites, when used individually or in combination, were found to be effective in improving the activation profiles of the clinically paralyzed or hypoactive muscles. Based on the MMR mapping and voluntary control mapping, four-site stimulation (C3–C4, C5–C6, C7–T1, T11–T12) was selected to allow effective activation of the upper extremity muscles and enabling trunk control required for effective upper extremity functions. 

### 3.4. Improved Activation of Upper Extremity Muscles Post ABRT + scTS Training

The participant completed 60 ABRT + scTS sessions with 100% compliance. Each block of ABRT + scTS session lasted for 2 h, 3–5 (4.3 average) days per week for 14 weeks. On average, each session lasted for approximately 60 min. 

**Figure 6:**
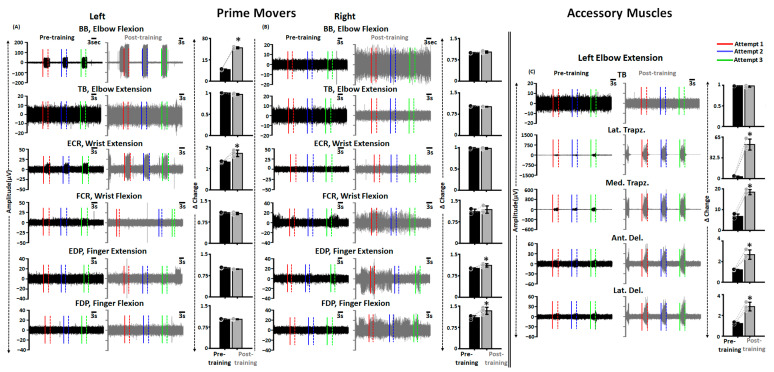
Improved primary mover and accessory muscles activation post-training. Shown are the EMG activity in various upper extremity muscles during FNPA engaging various primary mover muscles on the (**A**) left and (**B**) right side along with the bar graphs representing Δ change in EMG activity from the baseline during pre and post-training. (**C**) Shown are the EMG activity in various accessory upper extremity muscles during FNPA engaging left elbow extension along with the bar graphs representing Δ change in EMG activity from the baseline during pre and post-training. The solid and dashed line with the EMG plots represents the start and end of an attempt, respectively. Black and gray dots represent individual data points pre and post-training, respectively. * *p* < 0.05, Paired *t*-test. EMG: Electromyography; FNPA: Functional Neurophysiological Assessment; BB: Biceps Brachii; TB: Triceps Brachii; ECR: Extensor Carpi Radialis; FCR: Flexor Carpi Radialis; EDP: Extensor Digitorum Profundus; FDP: Flexor Digitorum Profundus; Lat. Trapz: Lateral Trapezius; Med. Trapz: Medial Trapezius; Ant. Del: Anterior Deltoid; Lat. Del: Lateral Deltoid.

As seen in Figure 6A, during pre-training FNPA on the left side, except BB and ECR, we did not observe robust activation of the primary movers. ABRT + scTS training resulted in significant improvements in the activation for the previously hypoactive muscles (BB and ECR), with no changes in the activation of the clinically paralyzed muscles (BB: t = −26.687, *p* < 0.001; ECR: t = −3.653, *p* = 0.001; TB: t = 3.354, *p* = 0.05; FCR: t = 1.372, *p* = 0.299; EDP: t = −0.277, *p* = 0.080; FDP: t = −0.277, *p* = 0.895, Paired *t*-test). In contrast, except FCR, we did not observe discernable activation of the upper extremity muscles during pre-training assessment on the right side. Post sixty training sessions, we observed increased activation for the EDP and FDP muscles with no changes in the activation levels of the remaining muscles (EDP: t = −2.968, *p* = 0.041; FDP: t = −2.504, *p* = 0.040; BB: t = −1.521, *p* = 0.271; TB: t = 1.565, *p* = 0.197; ECR: t = 0.146, *p* = 0.866; FCR: t = −0.695, *p* = 0.453, Paired *t*-test) (Figure 6B). Interestingly, the observed muscle activation post-training for the previously paralyzed muscles was not always time-locked to the provided audio cue, and the participant occasionally failed to relax muscles between consecutive attempts. Moreover, the extent of muscle activation varied between successive trials. 

For example, for right EDP muscle, the third attempt resulted in low-level muscle activation compared to the first attempt, no muscle activation was observed for the second attempt, and appreciable levels of muscle activation were observed during the rest period between the first and second attempts. However, muscle activation between two successive attempts was not seen for all muscles, and the video recording confirmed that the participant did not experience any spasmodic or autonomic dysfunction related symptoms during that time, indicating that the observed contractions during audio cue were indeed voluntary in nature.

As voluntary activation of the primary mover also results in the activation of distant muscles post SCI [46], we investigated the activation profiles of accessory muscles during selected upper extremity movements. Figure 6C shows the activation profiles of prime mover (TB) and accessory muscles (Lat. Trapz., Med. Trapz., Ant. Del., and Lat. Del.) during right elbow extension during pre and post-training FNPA. As seen in the figure, the activation levels were significantly greater for all selected accessory with no changes in the primary mover activation post-training (TB: t = 0.480, *p* = 0.775; Lat. Trapz.: t = −11.286, *p* < 0.001; Med. Trapz.: t = −36.190, *p* < 0.001; Ant. Del.: t = −6.032, *p* < 0.001; Lat. Del.: t = −8.795, *p* = 0.003, Paired *t*-test). 

### 3.5. Altered Spinal Cord Excitability Post ABRT + scTS Training

**Figure 7:**
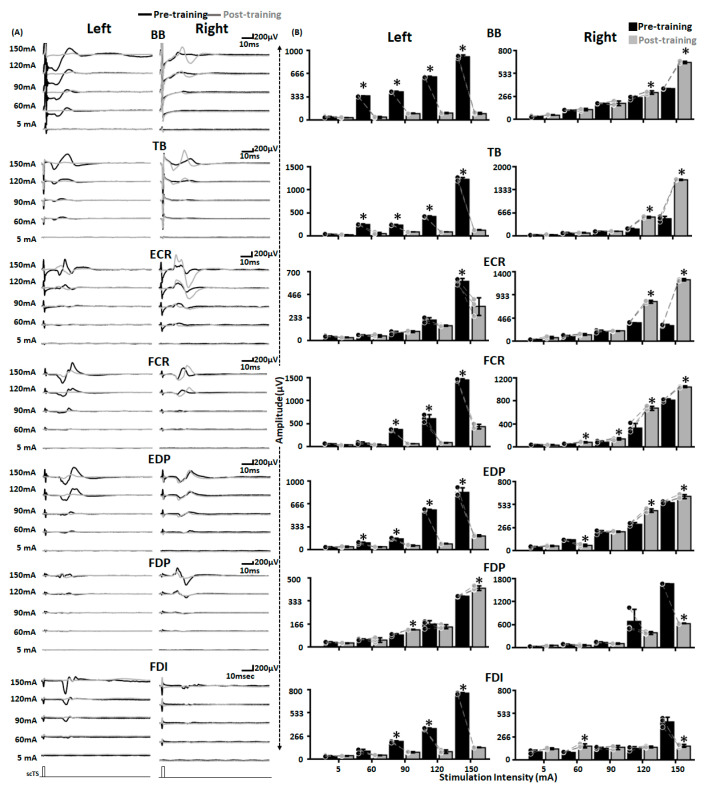
Altered spinal cord excitability post-training. Shown are (**A**) MMR (**B**) bar graphs representing raw peak-peak amplitude of MMR secondary to C5–C6 stimulation at various stimulation intensities from various upper extremity muscles on the left and right side pre (black) and post (gray) training. Black and gray dots represent individual data points pre and post-training, respectively. * *p* < 0.05, Paired *t*-test. EMG: Electromyography; MMR: Multisegmental Motor Responses; BB: Biceps Brachii; TB: Triceps Brachii; ECR: Extensor Carpi Radialis; FCR: Flexor Carpi Radialis; EDP: Extensor Digitorum Profundus; FDP: Flexor Digitorum Profundus; FDI: First Dorsal Interossei; scTS: Spinal Cord Transcutaneous Stimulation.

Shown here are the representative MMRs secondary to C5–C6 stimulation at varied stimulation intensities pre (black traces) and post (gray traces) training for tested upper extremity muscles (Figure 7A). We observed a reduction in MMR amplitudes post-training compared to the pre-training for the majority of the muscles on the left side. In contrast, for the right side, we observed an increase in MMRs amplitude post-training. When quantified for lower stimulation intensities that were equivalent to the intensities at which training was performed (60 and 90 mA), we observed a statistically significant decrease in P2P amplitudes for most of the upper extremity muscles on the left side. In contrast, for the right side, we observed a mix of increase (FCR at 60 and 90 mA, FDI at 90 mA), decrease (EDP at 60 mA, and FDP at 90 mA) or no change (BB, TB, ECR) in amplitudes post-training (Figure 7B and Table 1). Interestingly, at higher stimulation intensities (120 and 150 mA) that will allow possibly greater or maximum recruitment of the spinal neural circuitry, we observed a significant decrease or increase in the P2P amplitudes post-training compared to the pre-training amplitudes for most of the muscles on the left and right side, respectively. Unlike previous reports [23], we did not observe presence or emergence of long latency responses during the pre-training or post-training evaluation.

Table 1 Statistical data tabulated for MMR amplitudes from various upper extremity muscles pre and post-training. Paired *t*-test used for the analysis (*p* < 0.05). MMR: Multisegmental Motor Response; ABRT: Activity-based Recovery Training; scTS: Spinal Cord Transcutaneous Stimulation; BB: Biceps Brachii; TB: Triceps Brachii; ECR: Extensor Carpi Radialis; FCR: Flexor Carpi Radialis; EDP: Extensor Digitorum Profundus; FDP: Flexor Digitorum Profundus; FDI: First Dorsal Interossei; scTS: Spinal Cord Transcutaneous Stimulation.

### 3.6. Improved Sensations and Muscle Strength Post ABRT + scTS Training

**Figure 8:**
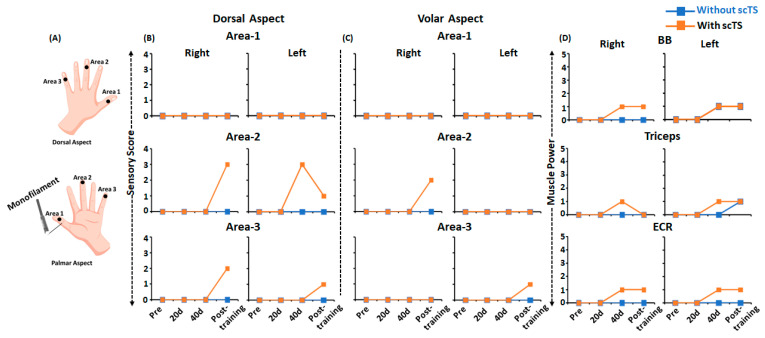
Sensory and power sub-component of the Graded Redefined Assessment of Strength, Sensation and Prehension (GRASSP) during pre, mid and post-training. (**A**) Schematic representing the areas on the dorsal and volar areas of the hand assessed using monofilament during sensory subscore on GRASSP assessment. Line plots representing (**B**,**C**) sensory subscore on the dorsal volar areas of the right and left hand and (**D**) muscle power subscore of the right and left upper extremity, during pre, mid and post-training with (orange) or without (blue) scTS. UE: Upper Extremity; scTS: Spinal Cord Transcutaneous Stimulation; BB: Biceps Brachii; ECR: Extensor Carpi Radialis; 20 d: 20 days training; 40 d: 40 days training.

Pre-training sensory assessment of the dorsal and volar aspects of the hand using monofilament (Figure 8A) during the GRASSP assessment revealed a complete absence of cutaneous sensations with or without multi-site scTS (score 0). Sensory assessment post 20, 40 and 60 ABRT sessions demonstrated improvements in sensory scores in areas 2 and 3, with no changes in area 1 (Figure 8B,C). Interestingly, improvement in sensory scores was only observed in the presence of scTS, with greater sensory improvements on the dorsal aspect of the hand. 

Similar to the sensory changes, we observed improvements in muscle strength of selected upper extremity muscles on the muscle power sub-component of the GRASSP assessment (Figure 8D). As seen, the selected upper extremity muscles (BB, TB, ECR) that scored zero (no palpable muscle contraction) during the pre-training assessment with and without scTS demonstrated improvements in muscle activity and obtained a score of one (palpable muscle contraction). Interestingly, changes in muscle strength were observed as early as 40 training sessions. However, similar to sensory changes, improvements in muscle strength were mostly observed in the presence of scTS, except for Left BB post 40 and 60 training sessions, and left TB post 60 training sessions, where similar improvements in the muscle strength were observed in the absence of scTS. No strength improvements were observed for the remaining upper extremity muscles (anterior deltoid, wrist extensors, extensor digitorum, opponens pollicis, flexor pollicis longus, finger flexors (DIII), finger abductors, and first dorsal interossei). 

### 3.7. No Changes in the Visually Appreciable Functional Performance Post ABRT + scTS Training

Despite improved activation of the clinically paralyzed or hypoactive muscle and improved sensations, we did not observe the presence of visually appreciable functional activity during GRASSP and NRS functional assessments with or without scTS.

### 3.8. Study-Related Adverse Effects

We observed skin-related issues, such as blistering or excess redness, following the initial few training sessions. The participant was referred to a dermatologist and in-house medical core that recommended regular breaks with no stimulation every 20 min during training sessions. We did not observe skin-related issues after implementing the suggested changes. The blood pressure ranged between 92 and 132 mm Hg for systolic and 54 and 95 mm Hg for diastolic. The heart rate ranged between 57 and 108 beats/min. No episode of autonomic dysfunction was observed or reported.

## 4. Discussion

The present case report demonstrates the therapeutic effectiveness of non-invasive multi-site spinal cord transcutaneous stimulation (scTS) targeting various spinal segments combined with activity-based recovery training (ABRT) in facilitating upper extremity sensory and motor recovery in an individual with severe cervical spinal cord injury (SCI) with minimal to no functional preservation below the injury level (AIS A, Neurological level of injury = C2). We leverage our scientific understanding of: (1) greater neuromodulatory effects of multi-site stimulation compared to a single-site [17,47]; (2) differential activation of upper extremity muscles depending on the stimulation site [13,48]; and (3) neuromodulatory effects of the lumbosacral spinal cord on the cervical spinal cord excitability [33,34] and trunk control [35,36] in facilitating upper extremity function. We hypothesized that multi-site scTS will effectively neuromodulate the spinal networks and will allow effective integration of ascending and descending information required for sensory and motor recovery post cervical SCI. Below we discuss the observed phenomenon, potential mechanisms for the observed improvements, and its clinical and therapeutic implications.

### 4.1. Activation of Upper Extremity Muscles with Targeted scTS Post Severe Cervical SCI

Despite the severity and extensiveness of the injury, scTS targeting spinal cord segments at or below the injury level resulted in robust multisegmental motor response (MMR) from various upper extremity muscles. Additionally, normalized recruitment curves and selectivity index indicate differential activation of upper extremity muscles based on scTS location. Although not entirely similar, the observed muscle activation was consistent with the myotome and topographical maps of the motoneuronal pool that spans across the cervical spinal cord in humans [45]. For example, greater activation of FDI muscle with motoneuronal pool spanning across C7–T1 was observed with scTS targeting C7–T1 spinal level compared to relatively proximal stimulation targeting C3–C4 and C5–C6 levels. To our surprise, similar to healthy adults [13], the distal muscles, such as ADM and FDI, demonstrated activation at higher stimulation intensity compared to their proximal counterparts (BB, TB, FCR). 

Similar selective activation of upper and lower limb muscles has been demonstrated in non-injured and injured adult rats [49,50], non-humans primates [44,51] and humans [13,48,52] secondary to invasive and non-invasive spinal cord stimulation targeting cervical spinal cord, respectively. The findings likely indicate the presence of viable segmental motoneurons and associated neural circuitry, such as dorsal root afferents, motor axons and interneuronal segments, innervating the upper extremity muscles that can be effectively engaged using scTS despite extensiveness of the cord damage.

### 4.2. Multi-Site scTS Induced Improved Sensory and Motor Activation Post Severe Cervical SCI

We hypothesized that scTS combined with ABRT will allow optimal neuromodulation of the spinal cord to a functional state due to activation of dormant pathways or improved activation potential of spared neural circuitry post injury [53,54], and facilitate re-emergence of supraspinal-spinal connectivity. In line with our hypothesis, we observed improvements in upper extremity voluntary muscle activation, muscle strength, and sensations. 

The immediate improvement in motor and sensory function in the presence of scTS indicates that the cervical spinal cord can be modulated into an optimal physiological state that allows greater and more effective supraspinal control over the sensory-motor network innervating the upper extremity. More specifically, scTS improves the activation threshold of the neural structures interacting with scTS, such as motoneurons, interneurons and motor axons [15,55], to a level that allows weak or residual descending pathways projecting to the spinal cord to activate them [34]. Moreover, sensory improvements in the presence of stimulation likely indicate that the sensory pathways can be modulated with scTS leading to better integration of the peripheral ascending inputs with descending volitional drive, ultimately resulting in improved motor task performance [56]. Indeed, improved sensory function is often correlated with motor recovery post SCI [57,58]. Similar immediate improvements in the motor and sensory function have been demonstrated previously with scTS targeting cervical and lumbar spinal cord in humans post SCI. For example, participants demonstrate immediate improvements in various hand functions, such as lateral pinch force, GRASSP strength and prehension score, in the presence of scTS compared to no scTS condition [22,23]. Similarly, compared to no scTS condition, participants demonstrate greater kinematic excursion of various lower limb segments during step-like locomotive behavior on the gravity-neutral device [16,17], improved ankle control [59], reduced lower limb spasticity [60], and improvements in the trunk control [36] in the presence of scTS. Overall, the obtained findings indicate the preserved neuroplastic abilities of the cervical spinal cord despite the severity of the injury and its intact receptiveness towards scTS.

### 4.3. Neurophysiological Basis of Sensory and Motor Recovery

Contrary to our expectation, we observed a decrease in the peak-peak amplitude of MMR for the majority of tested muscles at stimulation intensities at which ABRT was administered. A decrease in MMR amplitude indicates towards the reduced excitability of mono/di or polysynaptic components of the cervical spinal cord activated via scTS [15,55]. Similar decrease in the synaptic responses secondary to spinal cord stimulation combined with motor training has been reported previously in humans with SCI [61,62]. Furthermore, the increased activation of the inhibitory interneuronal pool may have contributed to the observed attenuation of MMR activity [63,64]. Reduced excitability of the neural structures interacting with scTS and greater activation of the inhibitory interneuronal circuitry potentially played a crucial role in reducing the spinal cord hyperexcitability which is often seen in individuals with SCI. These mentioned neurophysiological changes likely improved motor unit activation, leading to greater activation of the previously hypoactive or inactive muscles following training. Moreover, the presence of volitional EMG, even in the absence of scTS following sixty training sessions, indicates task specific re-organization of cervical spinal networks secondary to task-specific motor recovery training harnessing activity-dependent neuroplasticity [20,22,25]. Interestingly, improved activation was primarily seen for the relatively proximal muscle (BB, ECR, EDP, FDP) compared to the distally innervated muscles (ADM, FDI, TB), indicating towards the differential recovery in proximal-distal muscles as demonstrated previously [65]. Unlike previous reports, we did not observe long latency reflexes in MMR [23]. This may be due to insufficient remodeling of the intersegmental neural pathways responsible for the re-emergence of the polysynaptic responses and functional motor recovery [23]. 

### 4.4. No Changes in the Visually Appreciable Motor Functions

Despite a significant increase in the primary mover or accessory muscle activation, altered spinal cord excitability, and modest improvements in the sensation and muscle strength, the participant was unable to demonstrate any visually appreciable motor movement during the functional assessments post-training. The extensive damage to the spinal cord as evident secondary to the injury from MRI findings can partially explain the observed phenomena. Newer neuroimaging studies suggest limited sensorimotor recovery in individuals with extensive spinal cord damage [66,67]. Indeed, recent findings indicate that natural or scTS augmented motor recovery is more prominent in individuals with residual motor functions post cervical SCI [14,25,68]. Additionally, no prior involvement in the upper extremity rehabilitation program can provide further insight into the inability to demonstrate functional recovery post-training. Early involvement in task-specific motor training post injury is shown to be effective in facilitating motor recovery post-injury in humans [69,70]. Finally, although the participant underwent sixty sessions of ABRT combined with scTS, the administered total dosage might not be sufficient to result in functional recovery. Indeed, individuals with complete SCI demonstrate signs of lower limb functional recovery, such as independent stepping, after receiving more than 80–200 sessions of lumbosacral epidural stimulation along with locomotor training [6,7,71].

### 4.5. Clinical Implications and Recommendations: Identifying Opportunities for Recovery following Severe Cervical SCI

Recent quantitative descriptions of the sensorimotor recovery post SCI suggest that the recovery significantly depends on the severity and level of the injury. Moreover, cervical SCIs that account for more than fifty percent of the entire SCI population are associated with greater functional deficits and pose even greater challenges to medical and research accessibility [72]. Furthermore, cervical SCIs are often associated with poor upper extremity prognosis especially when injury results in complete loss of sensory and motor function below the injury level [73]. The complexity of the cervical spinal cord and its proximity to the vital brainstem structures poses significant challenges for implementing invasive therapeutic strategies [74], such as spinal cord epidural stimulation, that is known to be effective in recovering various physiological functions [6,7,71] encouraging the use of non-invasive neurostimulation techniques. However, reported use of non-invasive neuromodulation techniques, such as scTS, mostly involves individuals with residual arm and hand function post-injury making it challenging to draw supportive evidence for its therapeutic effectiveness in individuals with severe SCI with minimal to no sensory and motor preservation post injury [23,24]. The findings of the present work provide initial evidence of subthreshold multi-site scTS targeting systematically identified various spinal levels combined with ABRT effectiveness in facilitating sensory and motor recovery post cervical SCI. The findings extend the therapeutic potential of scTS to people with severe cervical SCI. However, the training may need to be extended beyond 60 sessions to obtain clinically meaningful functional improvements. The non-invasive nature of scTS and easy-to-use feature makes it a suitable neuromodulation device that can accelerate its translation to clinical practice for restoring upper extremity function post SCI. 

### 4.6. Limitation

As a case study, the presented work has certain inherent limitations. First, the data presented are only from one participant, and the findings may not be directly generalizable to a larger SCI population with severe injury. Second, the therapists involved in the ABRT and functional assessments were not blinded from the fact that the participant received scTS during motor training. Third, unlike previous studies, we added lumbosacral stimulation to further modulate the cervical spinal cord’s activity and to enable improved trunk control during ARBT. However, we did not comprehensively assess the individual effect of lumbosacral scTS on the truncal control. Nevertheless, the findings of the present study provide initial evidence that multi-site scTS along with ABRT facilitates sensory and motor recovery in individuals with severe SCI.

### 4.7. Conclusions

The presented work is a proof-of-principle of the therapeutic effectiveness of mult-site scTS targeting systematically identified various spinal segments combined with appropriately designed ABRT in promoting motor and sensory recovery in individuals with severe cervical SCI with minimal to no functional preservation below the injury level. Despite severity of injury, the spared or residual neural connections can be effectively modulated using neuromodulatory techniques, such as scTS. The present work extends the therapeutic effectiveness of scTS to individuals with severe cervical SCI. The current ongoing work in our research settings with a larger sample size and individuals with varied degrees of cervical SCI will further explore the exciting potential of scTS in restoring upper extremity function following a cervical SCI. The obtained information can be further exploited to guide or devise appropriate neuromodulation and rehabilitation programs, such as combinatorial stimulation techniques [75], robot-assisted training [76] or brain-computer interface controlled spinal stimulation [77,78], and will allow establishing neuromodulation as an effective and promising neuro-rehabilitative tool for individuals with severe cervical SCI resulting in debilitating sensory and motor dysfunctions.

## Figures and Tables

**Table 1:** MMR amplitudes from various upper extremity muscles pre and post-training.

Left								
	Muscle	BB	TB	ECR	FCR	EDP	FDP	FDI
Stimulation intensity (mA)	5	Pre: 47.37 ± 9.59	Pre: 44.90 ± 2.62	Pre: 44.47 ± 5.30	Pre: 61.91 ± 6.85	Pre: 38.22 ± 2.90	Pre: 35.60 ± 4.54	Pre: 36.91 ± 5.85
		Post: 37.20 ± 2.90	Post: 40.42 ± 2.19	Post: 33.28 ± 2.55	Post: 38.80 ± 7.44	Post: 46.94 ± 9.94	Post: 59.50 ± 1.10	Post: 39.82 ± 3.39
		t = 1.637 *p* = 0.243	t = 2.825 *p* = 0.085	t = 6.676 *p* = 0.021	t = 2.956 *p* = 0.097	t = −1.22 *p* = 0.344	t = 2.102 *p* = 0.170	t = −1.643 *p* = 0.241
	60	Pre: 344.27 ± 8.09	Pre: 254.8.10 ± 8.10	Pre: 54.21 ± 3.96	Pre: 82.11 ± 20.28	Pre: 99.98 ± 12.71	Pre: 55.80 ± 4.95	Pre: 93.01 ± 21.65
		Post: 43.16 ± 1071	Post: 59.00 ± 24.43	Post: 51.01 ± 11.31	Post: 83.71 ± 8.58	Post: 41.71 ± 3.66	Post: 51.30 ± 17.67	Post: 47.67 ± 2.40
		t = 29.891 *p* = 0.001	t = 20.575 *p* = 0.002	t = 0.746 *p* = 0.533	t = 3.465 *p* = 0.074	t = 11.134 *p* = 0.007	t = 0.348 *p* = 0.760	t = 3.541 *p* = 0.071
	90	Pre: 406.76 ± 5.46	Pre: 244.00 ± 11.38	Pre: 79.78 ± 14.84	Pre: 371.44 ± 17.42	Pre: 162.03 ± 9.21	Pre: 88.21 ± 8.07	Pre: 203.74 ± 10.04
		Post: 97.22 ± 3.72	Post: 90.97 ± 2.63	Post: 92.13 ± 7.66	Post: 66.99 ± 3.15	Post: 62.05 ± 8.00	Post: 125.85 ± 1.65	Post: 79.64 ± 5.62
		t = 127.519 *p* < 0.001	t = 21.243 *p* = 0.002	t = −0.952 *p* = 0.441	t = 25.779 *p* = 0.001	t = 84.052 *p* < 0.001	t = −6.712 *p* = 0.021	t = 13.726 *p* = 0.005
	120	Pre: 616.89 ± 6.60	Pre: 424.19 ± 12.43	Pre: 208.54 ± 24.42	Pre: 610.79 ± 78.94	Pre: 578.09 ± 11.05	Pre: 164.21 ± 28.19	Pre: 347.90 ± 6.58
		Post: 103.32 ± 5.99	Post: 90.83 ± 4.70	Post: 151.86 ± 7.25	Post: 89.23 ± 5.45	Post: 89.37 ± 6.77	Post: 146.63 ± 14.98	Post: 88.50 ± 16.70
		t = 154.236 *p* < 0.001	t = 41.959 *p* < 0.001	t = 3.483 *p* = 0.073	t = 11.011 *p* = 0.008	t = 60.323 *p* < 0.001	t = 1.371 *p* = 0.303	t = 27.120 *p* = 0.001
	150	Pre: 9.7.39 ± 20.44	Pre: 1225.64 ± 35.14	Pre: 598.14 ± 28.18	Pre: 1447.70 ± 18.76	Pre: 835.31 ± 59.45	Pre: 365.05 ± 2.40	Pre: 754.95 ± 9.89
		Post: 97.80 ± 8.94	Post: 131.08 ± 6.54	Post: 344.85 ± 87.60	Post: 439.89 ± 47.40	Post: 204.90 ± 12.36	Post: 423.32 ± 16.07	Post: 135.44 ± 3.21
		t = 73.577 *p* < 0.001	t = 51.915 *p* < 0.001	t = 4.721 *p* = 0.042	t = 38.843 *p* < 0.001	t = 16.119 *p* = 0.003	t = −6.966 *p* = 0.019	t = 123.062 *p* < 0.001
	Right							
Stimulation intensity (mA)	5	Pre: 33.13 ± 5.72	Pre: 34.01 ± 5.23	Pre: 38.66 ± 2.52	Pre: 41.85 ± 0.76	Pre: 40.84 ± 7.50	Pre: 34.73 ± 2.97	Pre: 47.52 ± 9.64
		Post: 51.59 ± 3.39	Post: 44.61 ± 2.97	Post: 71.35 ± 21.66	Post: 39.24 ± 1.90	Post: 55.95 ± 6.76	Post: 59.87 ± 8.85	Post: 64.96 ± 4.29
		t = −3.692 *p* = 0.066	t = −2.392 *p* = 0.139	t = −2.342 *p* = 0.143	t = 3.927 *p* = 0.060	t = −2.442 *p* = 0.134	t = −3.872 *p* = 0.060	t = −2.168 *p* = 0.162
	60	Pre: 106.23 ± 3.50	Pre: 88.21 ± 14.94	Pre: 102.60 ± 18.93	Pre: 55.51 ± 2.90	Pre: 126.72 ± 3.50	Pre: 82.69 ± 12.59	Pre: 45.34 ± 1.15
		Post: 112.77 ± 13.91	Post: 94.90 ± 4.58	Post: 135.15 ± 17.50	Post: 83.71 ± 8.58	Post: 60.60 ± 13.59	Post: 66.59 ± 16.81	Post: 80.94 ± 11.15
		t = −0.991 *p* = 0.426	t = −1.097 *p* = 0.386	t = −5.142 *p* = 0.035	t = −5.698 *p* = 0.029	t = 8.022 *p* = 0.015	t = 0.987 *p* = 0.439	t = −5.3983 *p* = 0.032
	90	Pre: 184.56 ± 2.48	Pre: 135.59 ± 5.57	Pre: 196.91 ± 22.99	Pre: 87.63 ± 15.16	Pre: 210.86 ± 20.75	Pre: 144.45 ± 16.45	Pre: 68.01 ± 6.29
		Post: 184.12 ± 26.10	Post: 140.53 ± 2.06	Post: 208.25 ± 9.41	Post: 140.09 ± 19.50	Post: 221.91 ± 6.80	Post: 110.15 ± 11.47	Post: 72.95 ± 10.70
		t = 0.026 *p* = 0.981	t = −1.123 *p* = 0.378	t = −0.654 *p* = 0.579	t = −5.232 *p* = 0.034	t = −0.893 *p* = 0.465	t = 2.145 *p* = 0.165	t = −1.114 *p* = 0.381
	120	Pre: 250.24 ± 16.99	Pre: 202.87 ± 13.14	Pre: 371.30 ± 6.93	Pre: 333.66 ± 76.91	Pre: 307.06 ± 16.37	Pre: 687.95 ± 304.14	Pre: 66.12 ± 10.11
		Post: 311.13 ± 18.37	Post: 546.70 ± 22.62	Post: 794.18 ± 27.13	Post: 671.82 ± 33.78	Post: 462.70 ± 21.52	Post: 385.10 ± 34.10	Post: 75.28 ± 5.62
		t = −29.052 *p* < 0.001	t = −52.371 *p* < 0.001	t = −29.667 *p* < 0.001	t = −13.450 *p* = 0.005	t = −11.514 *p* = 0.007	t = 1.661 *p* = 0.238	t = −2.055 *p* = 0.176
	150	Pre: 355.89 ± 3.15	Pre: 495.84 ± 73.21	Pre: 320.73 ± 13.61	Pre: 808.43 ± 20.18	Pre: 554.84 ± 11.29	Pre: 1655.22 ± 3.76	Pre: 217.69 ± 26.67
		Post: 660.49 ± 12.84	Post: 1615.98 ± 11.07	Post: 1230.59 ± 20.60	Post: 1040.21 ± 14.99	Post: 624.01 ± 22.71	Post: 631.28 ± 10.42	Post: 81.67 ± 7.33
		t = −45.088 *p* < 0.001	t = −26.140 *p* = 0.001	t = −49.095 *p* < 0.001	t = −14.543 *p* = 0.004	t = −10.444 *p* = 0.009	t = 125.105 *p* < 0.001	t = 7.710 *p* = 0.016

## Data Availability

The de-identified datasets generated through this study can be provided by the corresponding author upon reasonable request.

## Supplementary Materials

The following supporting information can be downloaded at: https://www.mdpi.com/article/10.3390/jcm12134416/s1, Figure S1: Iliac Crest vs. Clavicle anode placement and subthreshold stimulation, and FFT plots for ECR during wrist extension.
