# Supplementary material for: Multi-Site Spinal Cord Transcutaneous Stimulation Facilitates Upper Limb Sensory and Motor Recovery in Severe Cervical Spinal Cord Injury: A Case Study
Source: J Clin Med. 2023 Jun 30;12(13):4416. doi: 10.3390/jcm12134416 (PMC10342540; doi:10.3390/jcm12134416)
Supplement: Supplementary file 1 [file jcm-12-04416-s001.zip › jcm-2438100-supplementary.pdf]

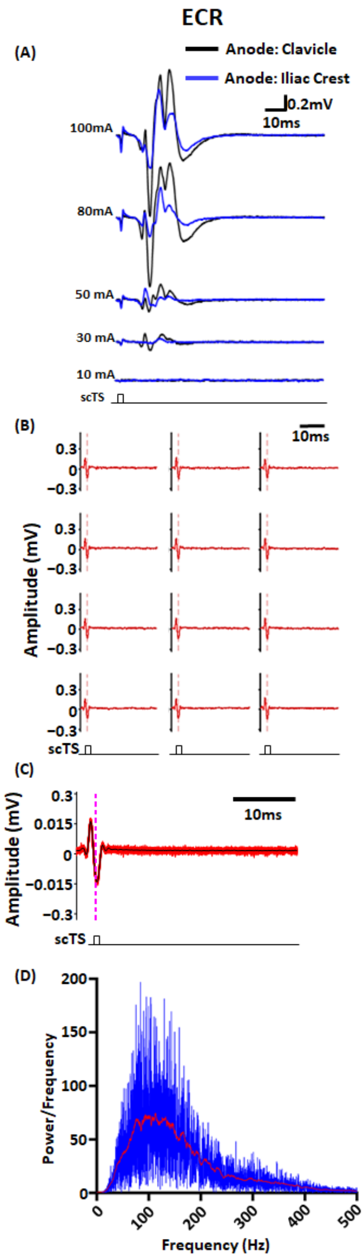

Supplementary Figure S1. Iliac Crest vs. Clavicle anode placement and subthreshold stimulation, and FFT plots for ECR during wrist extension. (**A**) Shown are the representative MMR traces secondary to C5-C6 stimulation from ECR muscle with anodes at clavicle (black traces) vs. iliac crests (blue traces) at varying stimulation intensities. Shown are (**B**) twelve individual representative MMRs and (**C**) mean (black trace) of fifty individual MMRs (red traces) secondary to C5-C6 stimulation from ECR muscle with 70 mA at 30 Hz. The pink dotted line represents the timepoint of scTS delivery. As seen, stimulation is subthreshold in nature resulting in no evoked responses from the upper extremity muscle. Note that waveforms observed in panel C and D that coincide with the scTS delivery time (pink dotted line) are stimulation artifacts. (**D**) Shown is the FFT plot associated with ECR activity during wrist extension without scTs in a neurologically intact individual. Red lines within FFT plots represent the moving mean of the obtained power at various frequencies. FFT: Fast Fourier Transform; ECR: Extensor Carpi Radialis; MMR: Multisegmental motor response; scTS: Spinal Cord Transcutaneous Stimulation.
